# A219 SHORT-TERM OUTCOMES OF UPADACITINIB IN PEDIATRIC IBD: A RETROSPECTIVE REVIEW

**DOI:** 10.1093/jcag/gwae059.219

**Published:** 2025-02-10

**Authors:** A Alharbi, L Rozka, S Lawrence, K Jacobson

**Affiliations:** Gastroenterology, Hepatology, and Nutrition, UBC, The University of British Columbia, Vancouver, BC, CA, academic, Vancouver, BC, Canada; Gastroenterology, Hepatology, and Nutrition, UBC, The University of British Columbia, Vancouver, BC, CA, academic, Vancouver, BC, Canada; Gastroenterology, Hepatology, and Nutrition, UBC, The University of British Columbia, Vancouver, BC, CA, academic, Vancouver, BC, Canada; Gastroenterology, Hepatology, and Nutrition, UBC, The University of British Columbia, Vancouver, BC, CA, academic, Vancouver, BC, Canada

## Abstract

**Background:**

Pediatric inflammatory bowel disease (IBD) requires innovative treatments to improve symptoms and reduce steroid use. Upadacitinib, a selective JAK-1 inhibitor, has shown efficacy in adults but pediatric data remain limited

**Aims:**

To assess clinical remission at 8 weeks in pediatric IBD patients treated with upadacitinib. Secondary aims include evaluating clinical response, steroid reduction, symptom improvement, biomarker changes, and adverse events

**Methods:**

This retrospective review included pediatric UC and CD patients treated with upadacitinib (45 mg daily for 8-12 weeks). Clinical remission was defined as wPCDAI/PUCAI ≤10 at 8 weeks. Secondary outcomes included clinical response (wPCDAI decrease >17.5 for CD, PUCAI ≥20 for UC), steroid reduction, symptom scores (abdominal pain, urgency, rectal bleeding, stool frequency), biomarkers (CRP, calprotectin), and adverse events. The Naranjo scale assessed adverse event probability

**Results:**

Seven patients (5 CD, 2 UC) with complete 8-week data were analyzed. Median age was 16 years (IQR: 14-17), with a 3:4 male-to-female ratio

**Clinical remission:**

At baseline, 4 patients were on both upadacitinib and steroids, and 3 on upadacitinib alone

At 8 weeks, 3/4 on steroids achieved remission, with 1 fully weaned

All 3 patients on upadacitinib alone achieved steroid-free remission

85.7% (6/7) achieved remission at 8 weeks, p = 0.01

33.3% (2/6) of remissions remained on steroids, and 66.7% (4/6) were steroid-free

**Secondary outcomes:**

**Clinical response:**

85.7% (6/7) achieved response, with wPCDAI/PUCAI scores improving from 40 (IQR: 30-52.5) to 5 (IQR: 0-15) CD scores improved from 52.5 (IQR: 40-60) to 7.5 (IQR: 0-25), and PUCAI from 32.5 (IQR: 25-40) to 0 (IQR: 0-0), p = 0.01

**Steroid reduction:**

4 patients were on steroids at baseline, with 1/4 fully weaned by 8 weeks

Baseline prednisone: 40 mg (IQR: 30-50) vs. 25 mg (IQR: 10-30) at 8 weeks, p = 0.02

**Symptom scores:**

Abdominal pain: 2 (IQR: 1-3) to 0 (IQR: 0-0), p = 0.03

Urgency NRS: 5 (IQR: 3-6) to 0 (IQR: 0-2), p = 0.01

Rectal bleeding: 2 (IQR: 0-3) to 0 (IQR: 0-0), p = 0.04

Stool frequency: 2 (IQR: 1-3) to 0 (IQR: 0-1), p = 0.02

**Biomarker response:**

CRP: 25 mg/L (IQR: 3.1-39) to 10 mg/L (IQR: 2-18), p = 0.03

Calprotectin: 2000 µg/g (IQR: 580-5449) to 800 µg/g (IQR: 74-1378), p = 0.04

**Adverse events:**

71.4% (5/7) experienced acne, 28.6% (2/7) headaches, and 28.6% (2/7) nausea

No serious adverse events were reported. Median Naranjo score: 2

**Conclusions:**

Upadacitinib improved symptoms and reduced steroid use in pediatric IBD, with 85.7% (6/7) achieving remission at 8 weeks. Of these, 66.7% (4/6) achieved steroid-free remission. Clinical response was achieved in 85.7% (6/7), with significant improvements in both UC and CD. Mild adverse events were reported, supporting further exploration of upadacitinib in pediatric IBD. Additional patients will be recruited in the coming months to expand this dataset

**Funding Agencies:**

None

